# Integrated Multi-Omics Analysis Reveals Molecular Signatures Associated with Growth Divergence in Mandarin Fish (*Siniperca chuatsi*)

**DOI:** 10.3390/ijms27198578

**Published:** 2026-09-25

**Authors:** Ifeanyi Solomon Ahamba, Lionel Kinkpe, Xin Zhang, Xiaoli Yao, Christian Uchechukwu Ekugba, Hongxia Hu, Ran Cui

**Affiliations:** 1 National Engineering Research Center for Freshwaters (Beijing), Beijing Key Laboratory of Fisheries Biotechnology, Fisheries Science Institute, Beijing Academy of Agriculture and Forestry Sciences, Beijing 100068, China; 2 College of Animal Science and Technology, Northwest A&F University, Yangling, Xianyang 712100, China; 3 National Demonstration Center for Experimental Fisheries Science Education, Shanghai Ocean University, Shanghai 201306, China; 4 Department of Animal Science, Nnamdi Azikiwe University, Awka 420110, Anambra State, Nigeria; cu.ekugba@unizik.edu.ng

**Keywords:** *Siniperca chuatsi*, growth divergence, skeletal muscle, proteasome, oxidative phosphorylation, haemoglobin

## Abstract

Within-cohort growth heterogeneity constrains aquaculture productivity, and its molecular correlates in carnivorous teleosts are poorly described. We compared the three heaviest and the three lightest individuals (n = 3 biological replicates per group, one fish per replicate) from a single cohort of mandarin fish (*Siniperca chuatsi*) at 90 days post-hatch, which differed 3.09-fold in body mass (*p* = 7.1 × 10^−5^), by combining muscle RNA sequencing with label-free quantitative proteomics of muscle and liver. Muscle transcriptomics identified 827 differentially expressed genes. The proteasome (ko03050) was the only KEGG pathway reaching significance, with all 14 contributing subunits being more abundant in slow-growing fish, and GO enrichment recovered proteasome, peptidase and endopeptidase complexes alongside serine-type peptidase activities in the same direction. Proteomics identified 37 differentially abundant proteins in muscle and 24 in liver at a nominal threshold. Protein-level gene set enrichment analysis showed negative enrichment of oxidative phosphorylation (NES = −1.96, adjusted *p* = 0.001) and of mitochondrial proteins (NES = −1.77, adjusted *p* = 0.005) in fast-growing muscle. Haemoglobin subunits were more abundant in fast-growing muscle and liver at the protein level, while muscle haemoglobin transcripts showed paralog-specific differences. The cross-sectional extreme-phenotype design identifies molecular signatures associated with the observed growth phenotype without establishing causality. The dataset provides candidate genes and proteins for functional study in *S. chuatsi*.

## 1. Introduction

Global aquaculture production now exceeds capture fisheries as the primary source of fish for human consumption, and continued improvement in the growth efficiency of farmed species is required to meet demand projections [1]. Within-cohort growth heterogeneity is one obstacle to that goal: under uniform feeding and rearing conditions, individual fish of the same age and cohort origin can differ several-fold in body mass, which raises grading costs, extends production cycles and reduces yield uniformity [2,3].

Mandarin fish (*Siniperca chuatsi*) are among China’s most commercially valuable freshwater carnivores. Size divergence is pronounced during the juvenile period, and both piscivorous feeding behaviour and the early establishment of dominance hierarchies in captive rearing contribute to it [4]. Physiological, transcriptomic and genomic aspects of *S. chuatsi* biology have received attention [5,6,7], and muscle transcriptomics has been applied to individuals of contrasting body size in this species [8]. Quantitative proteomics has not been combined with transcriptomics in this context, and no study has profiled muscle and liver together in fish drawn from the two extremes of a single growth distribution. The absence of protein-level data is a practical limitation because transcript abundance is an imperfect predictor of protein abundance in eukaryotes [9]. Integrating proteomic information can therefore provide complementary evidence for molecular features associated with growth phenotype.

Somatic growth depends on net muscle protein accretion, which reflects the balance between protein synthesis and degradation. The ubiquitin-proteasome system carries out selective intracellular proteolysis, degrading ubiquitin-tagged substrates and recycling their amino acids [10,11,12]. Proteasomal gene expression in teleosts is responsive to nutritional status and growth-related physiological variation [13]. Energy substrate use is a second axis: rapid growth in teleost skeletal muscle is accompanied by preferential hypertrophy of glycolytic fibres, which carry lower mitochondrial density than oxidative fibres and correspondingly lower oxidative phosphorylation capacity per unit mass [14,15]. Haemoglobin transcripts and proteins have also been reported in non-erythroid teleost tissues, including skeletal muscle, under conditions of elevated oxygen demand [16,17], although the cellular origin of such signals in bulk tissue is difficult to establish.

The present study combined muscle RNA sequencing with label-free quantitative proteomics of muscle and liver in fast-growing and slow-growing individuals from the same rearing cohort. The objectives were to identify genes and proteins that differ between the two growth phenotypes; to determine whether proteolytic, bioenergetic and oxygen-handling signals appear together in the same animals; and to assess how far signals detected in one molecular layer or tissue are recovered in the other.

## 2. Results

### 2.1. Phenotypic Characterisation

Fast-growing fish weighed 71.0 ± 4.6 g, while slow-growing fish weighed 23.0 ± 1.7 g (mean ± SD), a 3.09-fold difference (*p* = 7.1 × 10^−5^). Body length was 16.53 ± 0.99 cm versus 10.40 ± 0.40 cm, a 1.59-fold difference (*p* = 5.8 × 10^−4^; Figure 1). The FM and SM groups represented the upper and lower extremes of the observed cohort size distribution, respectively.

### 2.2. Transcriptomic Differentiation Between Growth Phenotypes

Sequencing produced 45.6–48.6 million raw reads per sample, with a clean read retention of 96.2–98.3%, Q30 above 97.1% and comparable GC content across libraries (Table S1, Figure S1). Principal component analysis of global expression separated FM and SM samples without overlap, PC1 and PC2 accounted for 47.20% and 19.56% of total variance (Figure 2a), and hierarchical clustering of differentially expressed genes reproduced the same grouping (Figure 2b). A total of 827 genes met the differential expression criteria, 318 with higher abundance in FM and 509 with higher abundance in SM (Figure 2c). Complete per-gene results, including baseMean, standard error, Wald statistic, raw and adjusted *p* values and annotation for all 29,908 features, are provided in Table S2.

### 2.3. Proteasomal and Peptidase Transcripts Are More Abundant in Slow-Growing Muscle

KEGG over-representation analysis of the 827 differentially expressed genes returned a single significant pathway, the proteasome (ko03050; gene count = 14; adjusted *p* = 2.85 × 10^−6^; Figure 3a). All 14 contributing subunits (*Psmc3*, *Psmc4*, *Psmc5*, *Psma1*, *Psma2*, *Psma5*, *Psma7*, *Psmb3*, *Psmd1*, *Psmd2*, *Psmd11b*, *Psmd12*, *Psmd13* and *Psmd14*) were more abundant in slow-growing fish, with no subunit showing the opposite direction. Across the dataset, 18 proteasome-annotated genes reached the differential expression threshold, and all 18 were higher in SM, with log_2_ fold changes between −1.26 and −2.04.

GO enrichment returned ten significant terms, five in the Cellular Component category and five in Molecular Function. The cellular component terms were peptidase complex (count = 6; adjusted *p* = 0.044), proteasome core complex (count = 5; adjusted *p* = 0.044), proteasome complex (count = 5; adjusted *p* = 0.044), endopeptidase complex (count = 5; adjusted *p* = 0.044) and extracellular space (count = 5; adjusted *p* = 0.044). None of the genes contributing to the four proteolytic complex terms was more abundant in fast-growing fish. The molecular function terms were serine-type endopeptidase activity (count = 17; adjusted *p* = 2.48 × 10^−4^), serine-type peptidase activity and serine hydrolase activity (count = 17 each; adjusted *p* = 7.28 × 10^−4^), endopeptidase activity (count = 24; adjusted *p* = 7.28 × 10^−4^) and peptidase activity (count = 29; adjusted *p* = 0.035). The first three nested GO terms shared the same 17 contributing genes. Genes contributing to these terms were predominantly more abundant in slow-growing fish (15 of 17 for the serine-type terms, 20 of 24 for endopeptidase activity, 24 of 29 for peptidase activity). A total of 24 unique genes contributed across the four serine-type and endopeptidase terms. Genes recurring across all four included *Cela2a*, *Cela3b*, *Cfb*, *Plg*, *Prep*, *Prss1*, *Prss12*, *Prss21*, *Tmprss2* and *Tmprss6*, together with the annotation entries *Elastase-1*, *Trypsin-1*, *Trypsin-3* and *Chymotrypsin A*, while *Zmpste24*, *Hceb* and *Adam11* each contributed to a single term.

A protein–protein interaction network for the 827 differentially expressed genes was constructed in STRING using *Danio rerio* as the reference species (Figure 3b). The network comprised 156 nodes and 261 edges with interaction confidence scores from 702 to 999 (mean 907); 169 of 261 edges (65%) scored ≥ 900 and 40 reached 999. Thirty-one connected components were resolved, indicating that interactions were distributed across discrete modules rather than one continuous network. Two components dominated. The first contained 26 nodes and 56 edges (mean score 897) with *SF3A2* (degree = 13), *SF3B4* (degree = 11) and *snrpd3* (degree = 10) as hubs. The second contained 19 nodes and 112 edges, an edge density of 65% with a mean interaction score of 970; *PSMC3* was the most connected node in the entire network (degree = 18), followed by *PSMA7*, *PSMA5*, *PSMD11B* and *PSMB3* (degree = 16 each). This component corresponds to the proteasome signal recovered by KEGG and GO enrichment. The remaining 29 components contained 2–11 nodes with lower internal connectivity.

### 2.4. Proteomic Profiling of Muscle and Liver

Across the 12 proteomic samples (3 FM muscle, 3 SM muscle, 3 FM liver, 3 SM liver), 380 proteins were identified from 3938 peptides at a false-discovery rate below 1%. Within-group Pearson correlations were 0.829 (FM muscle), 0.833 (SM muscle), 0.765 (FM liver) and 0.612 (SM liver), with coefficients of variation of 23.7%, 28.3%, 24.9% and 48.5%; the SM liver group was the most variable (Figure 4a). Principal component analysis identified tissue as the dominant source of variance (PC1 = 70.8%), with partial separation of phenotypes on PC2 (5.6%; Figure 4b). At an exploratory, uncorrected threshold of *p* < 0.05, 37 proteins were classified as differentially abundant in muscle (18 higher and 19 lower in FM) and 24 in liver (9 higher and 15 lower in FM; log_2_ fold change −1.79 to 1.92; Figure 4c–e).

Hierarchical clustering of muscle proteins resolved two clusters (Figure 5). The cluster of 18 proteins higher in FM was dominated by haemoglobin subunits, including Q2KKV1 (log_2_FC = 2.731, *p* = 0.011), Q2KKV2 (log_2_FC = 2.725, *p* = 0.016), A0EM53 (log_2_FC = 2.44, *p* = 0.015) and Q2KKV0 (log_2_FC = 2.44, *p* = 0.008), together with myeloid leukaemia factor 1 (A0A165R1Y8). The cluster of 19 proteins lower in FM included parvalbumin G8GWA4 (log_2_FC = −3.57, *p* = 0.024), ATP synthase subunit 8 F8UZB7 (log_2_FC = −1.48, *p* = 0.004), cytochrome c oxidase subunits Q2KKX8 (log_2_FC = −1.04, *p* = 0.002) and Q2KKX9, and carnitine O-palmitoyltransferase 1 A0A165R1H5. In liver, haemoglobin subunits Q2KKV2 (log_2_FC = 1.92, *p* = 0.007), Q2KKV1 (log_2_FC = 1.91, *p* = 0.015) and Q2KKV0 (log_2_FC = 1.77, *p* = 0.015) and LECT2 A0A5J6E2E9 (log_2_FC = 1.73, *p* = 0.017) were the most strongly elevated in FM fish, while PNP A0A5J6E2H0 (log_2_FC = −1.79, *p* = 0.0009), cryptochrome 1 A0A0G2UK93 (log_2_FC = −1.69, *p* = 0.003) and nucleobindin 1 A0A8G0YKN5 (log_2_FC = −1.61, *p* = 0.035) were the most strongly reduced (Figure S2).

### 2.5. Oxidative Phosphorylation and Mitochondrial Proteins Are Lower in Fast-Growing Muscle

Protein-level GSEA in muscle returned two significant KEGG terms, oxidative phosphorylation (NES = −1.961, *p* < 0.001, adjusted *p* = 0.001) and cardiac muscle contraction (NES = −1.801, *p* = 0.002, adjusted *p* = 0.017), both negatively enriched in FM fish (Figure 6a). Subcellular GSEA returned one significant term, mitochondrial proteins (NES = −1.774, *p* = 0.004, adjusted *p* = 0.005), also negatively enriched in FM muscle. No muscle GO GSEA term reached adjusted *p* < 0.05; the five terms with the largest |NES| were ribosome (NES = 1.018), structural constituent of ribosome (NES = 0.994), translation (NES = 0.994), protein phosphorylation (NES = 0.828) and protein kinase activity (NES = 0.828) (Figure S3a,b). No liver KEGG, GO or subcellular term reached adjusted *p* < 0.05; the five liver KEGG terms with the largest |NES| were focal adhesion (NES = −1.618), adrenergic signalling in cardiomyocytes (NES = −1.548), cytoskeleton in muscle cells (NES = −1.524), autophagy-animal (NES = −1.426) and cardiac muscle contraction (NES = −1.372) (Figure 6b).

Subcellular annotation of the differentially abundant proteins placed approximately 35% of muscle proteins in mitochondria and 25% in cytoplasm, consistent with the GSEA signal, while liver proteins were distributed across cytoplasm (~38%), nucleus (~30%), mitochondria (~20%) and extracellular space (~12%) (Figure 6c,d). The muscle protein–protein interaction network formed a densely connected core around F8UZB6, Q2KKX0, Q2KKX9 and A0A0G2T6D8, with proteins of both directions interspersed and no directional segregation (Figure 7).

### 2.6. Cross-Tissue Overlap of Differentially Abundant Proteins

Of the 61 differentially abundant proteins (37 muscle, 24 liver), 6 were shared between tissues (Q2KKV0, Q2KKV1, Q2KKV2, A0EM53, F8UZB6, F8UZC2), and all 6 were higher in FM fish in both tissues (Table S2). Thirty-one were specific to muscle and eighteen to liver. K-means clustering across the four sample groups resolved four expression subclusters: higher in both slow groups; higher in both fast groups; liver-enriched irrespective of phenotype; and muscle-enriched irrespective of phenotype.

### 2.7. Transcript-Protein Correspondence in Muscle

Entity-level matching between the muscle transcriptome and proteome resolved twelve transcript–protein pairs involving six protein accessions (Table S3). One pair was an exact gene-symbol match, six were paralog or isoform matches within a gene family, and five were description-based. Five of the twelve pairs agreed in direction and seven did not.

Haemoglobin accounts for most of the ambiguity. Six haemoglobin genes were differentially expressed in muscle, and they split by paralog: *hbab* (log_2_FC = 2.16, adjusted *p* = 4.8 × 10^−4^), an *hbb1*-like locus (log_2_FC = 2.01, adjusted *p* = 1.4 × 10^−5^) and *hbaa* (log_2_FC = 1.83, adjusted *p* = 1.5 × 10^−3^) were higher in fast-growing fish, while *hba1* (log_2_FC = −1.60, adjusted *p* = 4.2 × 10^−5^), *hbb2* (log_2_FC = −1.86, adjusted *p* = 1.2 × 10^−4^) and a second *hbb1*-like locus (log_2_FC = −1.24, adjusted *p* = 0.016) were higher in slow-growing fish. The corresponding proteins are annotated as haemoglobin alpha-A and beta-A chain fragments and cannot be assigned to individual paralogs, so the transcript–protein relationship for haemoglobin is one to many, and its directional interpretation depends on which paralog is paired with which protein. The protein-level increase in fast-growing muscle and liver is consistent across the four haemoglobin accessions detected; the transcript-level pattern is paralog-specific and is not a uniform increase (Figure 8a).

The remaining pairs behaved similarly. Three myosin heavy chain transcripts were differentially expressed in opposite directions (log_2_FC = 3.12, −1.70 and −1.51), and all three map by description to the single fast skeletal myosin heavy chain protein G0YU50 (log_2_FC = 0.57, *p* = 0.003), so one pair agrees in direction and two do not. Two cytochrome c oxidase transcripts, subunit 4 isoform 2 (log_2_FC = 1.31) and subunit 6A (log_2_FC = −1.33), both map to Q2KKX8 (subunit 7A2; log_2_FC = −1.04, *p* = 0.001), giving one agreement and one disagreement within the same complex. *Ptk2b* was more abundant in slow-growing muscle at the transcript level (log_2_FC = −3.16, adjusted *p* = 0.040), while the matched protein C7ATY9 was higher in fast-growing muscle (log_2_FC = 7.40, *p* = 5.1 × 10^−7^) (Figure 8a).

Among the 35 protein identifiers that could be mapped to gene symbols, one corresponded directly to a differentially expressed gene (Figure 8b). Several protein identifiers mapped to multiple *S. chuatsi* transcripts or paralogous genes, limiting direct one-to-one transcript–protein correspondence (Figure 8a). Pathway-level integration nevertheless identified shared molecular patterns in muscle, including oxidative phosphorylation and haemoglobin-associated features.

## 3. Discussion

Muscle transcriptomics and muscle and liver proteomics in fish from the two ends of a single growth distribution identified three signals: coordinated elevation of proteasomal and peptidase transcripts in slow-growing muscle, reduced oxidative phosphorylation and mitochondrial protein abundance in fast-growing muscle, and higher haemoglobin protein abundance in both tissues of fast-growing fish. These molecular patterns characterize the contrasting growth phenotypes observed within the cohort.

### 3.1. Proteolytic Gene Expression and Growth Phenotype

The proteasome was the only KEGG pathway to reach significance in the muscle transcriptome, and every contributing subunit was more abundant in slow-growing fish. The GO results point the same way, with proteasome core, proteasome, peptidase and endopeptidase complexes all enriched and none of their member genes elevated in fast-growing fish. The proteasome subunits also form the densest module of the interaction network, with *PSMC3* as its most connected node. KEGG and GO enrichment, together with the protein–interaction network, consistently highlighted proteasome-associated genes.

Elevated expression of the degradative machinery in the smaller animals is consistent with what is known about proteostasis in teleost muscle. The 26S proteasome executes selective intracellular proteolysis and recycles amino acids from ubiquitin-tagged substrates [10,11,12], and its subunit expression in fish tracks nutritional status: fasting reduces and re-feeding restores proteasomal activity in gilthead sea bream [13]. Net protein accretion is the difference between synthesis and degradation, and a shift in that balance towards degradation is the conventional molecular correlate of restricted growth in vertebrate muscle [10,18]. In 2020, Liu et al. reported the complementary observation in this same species, finding protein synthesis, ribosome, and energy metabolism transcripts elevated in smaller three-month-old *S. chuatsi* relative to larger individuals [8], which suggests that both arms of protein turnover are transcriptionally more active in the slower-growing phenotype.

The biological basis of the elevated proteolytic transcriptional profile remains unresolved. Fish were maintained under communal feeding conditions, and individual feed intake was not recorded. Reduced feed access arising from social hierarchy, nutritional state at the time of sampling, and intrinsic differences in growth regulation would all be expected to leave a signature in proteolytic gene expression, and the present design does not distinguish among them. Proteasome subunit transcript abundance is also an imperfect proxy for proteolytic flux since assembly and activity are regulated post-transcriptionally; direct measurement of proteasome activity would be required to establish that degradative capacity differs.

These genes include digestive and coagulation-associated proteases such as *Prss1*, *Cela2a* and *Plg*, indicating that the peptidase enrichment extends beyond canonical intracellular proteolysis.

### 3.2. Mitochondrial and Oxidative Phosphorylation Proteins in Fast-Growing Muscle

Protein-level GSEA identified significant negative enrichment of oxidative phosphorylation, cardiac muscle contraction and mitochondrial protein categories in fast-growing muscle after multiple-testing correction. Individual proteins in the same direction include ATP synthase subunit 8, two cytochrome c oxidase subunits and carnitine O-palmitoyltransferase 1, the rate-limiting enzyme for mitochondrial long-chain fatty acid import. Lower oxidative capacity in the faster-growing animals is at first sight counterintuitive.

One established explanation in teleosts is fibre composition. Rapid somatic growth in teleost muscle is associated with hypertrophy of glycolytic white fibres, which have lower mitochondrial density than oxidative fibres [14,15]. The reduced abundance of oxidative phosphorylation proteins observed in FM muscle is consistent with this metabolic organization. Lower carnitine O-palmitoyltransferase 1 abundance further indicates differences in mitochondrial fatty-acid utilization [19]. These results support a distinct mitochondrial and energy-metabolic profile in the muscle of the two growth phenotypes. Reduced expression of respiratory chain components has been reported in teleosts under other divergent physiological states, including albino and normally pigmented Wels catfish [20], so the association between oxidative phosphorylation and a particular phenotype is not by itself specific to growth.

### 3.3. Haemoglobin Signals in Muscle and Liver

Four haemoglobin accessions were more abundant in fast-growing fish in both muscle and liver, and they are among the largest protein-level differences in the dataset. At the transcript level the picture is more structured: three globin loci were higher in fast-growing muscle and three were higher in slow-growing muscle, so the muscle transcriptome shows a shift in the relative representation of globin paralogs rather than a general increase. The protein identifications are fragments annotated as alpha-A and beta-A chains and cannot be assigned to individual paralogs, which limits how far the two layers can be compared for this gene family.

The concordant increase in haemoglobin proteins in FM muscle and liver indicates a systemic association between haemoglobin abundance and growth phenotype. Globin transcripts were also detected in muscle, although their direction differed among paralogs. Haemoglobin expression has been reported in non-erythroid teleost tissues under increased oxygen demand [16,17], while residual erythrocytes may also contribute to globin signals in bulk-tissue preparations. The cellular origin and physiological significance of the haemoglobin-associated pattern remain to be established.

### 3.4. Correspondence Between Molecular Layers

Entity-level overlap between the two molecular layers was limited, with one exact gene-symbol match among 35 resolvable protein identifiers. Among the 12 transcript–protein pairs identified, 5 showed concordant directions of change and 7 were discordant. Moderate correlation between mRNA and protein abundance is a general feature of eukaryotic systems, attributable to translational regulation and differences in protein stability [9,21], and transcriptomics and proteomics are commonly complementary rather than redundant in fish [22]. The limited entity-level correspondence is consistent with the moderate relationship commonly observed between transcript and protein abundance [9,21]. In the present dataset, additional complexity arose from one-to-many relationships involving globins, myosin heavy chains and cytochrome c oxidase subunits. Despite limited direct correspondence, pathway-level integration identified shared molecular patterns across the transcriptomic and proteomic datasets.

Previous transcriptomic studies of growth variation in mandarin fish have primarily implicated fatty-acid biosynthesis, cell-cycle regulation and the IGF system [23,24]. The present multi-omics analysis additionally identified proteasome-associated transcriptional differences and a protein-level oxidative phosphorylation signature.

### 3.5. Limitations of the Study

The study used three biological replicates per phenotype and an extreme-phenotype design to characterize molecular differences between individuals at opposite ends of the cohort size distribution. Validation in larger independent cohorts will be important for estimating the population-level consistency and magnitude of these associations.

The cross-sectional design captures molecular differences after growth divergence was established and does not resolve their temporal sequence. Fish originated from a single shared rearing environment, with communal feeding and no individual measurements of feed intake or dominance behaviour. Future longitudinal and replicated-cohort studies could determine how these factors contribute to the molecular signatures identified here. Furthermore, individual protein-level differences were identified at nominal *p* < 0.05, while pathway-level GSEA significance was assessed using Benjamini–Hochberg-adjusted *p* values. Independent experimental validation will be required to establish the functional relevance of the proteasome, oxidative phosphorylation and haemoglobin-associated signatures. In particular, proteasome activity, muscle fibre composition and the cellular localisation of haemoglobin represent priorities for targeted follow-up studies.

Direct transcriptome–proteome integration was restricted to muscle because transcriptomic profiling was not performed in liver. Functional annotation depended on cross-species orthology assignment to *Danio rerio* and on UniProt records from related teleosts, which introduces uncertainty at the level of individual gene and protein assignments, particularly within multi-member families. The conclusions apply to skeletal muscle and liver; other tissues, including brain, intestine and endocrine organs, may carry different molecular signatures of the same phenotype.

## 4. Materials and Methods

### 4.1. Experimental Animals, Cohort and Phenotype Selection

Mandarin fish were obtained from the Bashaxinquan Aquaculture Farm (Beijing, China). All fish originated from a single artificial spawning of unrelated broodstock and were reared together in one indoor cement tank holding more than 100 individuals, at 22 °C under continuous aeration. Ice-fresh silver carp were offered twice daily, once in the morning and once in the evening, delivered gradually in small quantities over approximately 30 min per feeding session. Approximately one-third of the tank water was renewed at each water exchange, performed about once per week. Individual feed intake was not recorded, and fish were not individually tagged because of their small size at the time of the experiment.

At 90 days post-hatch, all fish were weighed and measured. This was the only measurement point for the present study. The three heaviest individuals were assigned to the fast-growing group (FM) and the three lightest to the slow-growing group (SM); selection was therefore based on the extremes of the observed size distribution rather than on a predefined percentile or weight cut-off. Six fish were profiled in total, three per phenotype, and each fish constituted one biological replicate for both transcriptomic and proteomic analysis. Sex was not recorded: sexual differentiation in *S. chuatsi* is not complete before approximately three months post-hatch, so reliable classification was not possible at sampling, and sex was not treated as a biological factor.

Skeletal muscle and liver were selected for molecular profiling. White epaxial muscle is the dominant contributor to somatic mass and the site at which protein turnover, mitochondrial content and fibre composition act on growth; liver integrates nutrient processing, intermediary metabolism and endocrine output and provides a systemic counterpart to the local muscle phenotype. Other tissues, including brain, intestine and endocrine organs, were not examined, and may show different patterns.

All animal procedures were approved by the Institutional Animal Care and Use Committee of the Beijing Academy of Agriculture and Forestry Sciences (Protocol No. BAAFS-KJLL-20260332) and were conducted in accordance with institutional guidelines for the ethical use of experimental animals. Efforts were made to minimise the number of animals used and any potential suffering.

### 4.2. Anaesthesia and Tissue Collection

Fish were transferred individually to an anaesthetic bath containing clove oil (eugenol content ≥ 72%; Sigma-Aldrich, St. Louis, MO, USA) dissolved in ethanol (1:9 *v*/*v* stock) and diluted to 60 mg/L in system water at 22 °C [25]. Induction was confirmed by loss of equilibrium and cessation of spontaneous opercular movement, typically within 2–3 min, after which fish were removed from the bath, weighed and measured. Euthanasia was performed by anaesthetic overdose in a 200 mg/L clove oil bath until opercular movement ceased completely. White epaxial muscle and liver were excised on ice within 2 min, snap-frozen in liquid nitrogen and stored at −80 °C. Each biological replicate consisted of tissue from a single individual.

### 4.3. RNA Extraction and Transcriptome Sequencing

Total RNA was extracted from muscle of each individual separately using TRIzol-based homogenisation; no samples were pooled, and each of the six libraries derives from one fish. RNA integrity and purity were assessed by spectrophotometry and capillary electrophoresis, and samples with an RNA integrity number ≥ 7.0 and OD_260/280_ between 1.8 and 2.0 were used. Messenger RNA was enriched with poly-T oligo-attached magnetic beads, fragmented, and converted to first-strand cDNA using random hexamer primers, followed by second-strand synthesis. Non-strand-specific libraries were prepared by end repair, A-tailing, adapter ligation, size selection, PCR amplification and purification, then quantified by Qubit fluorometry and quantitative PCR and checked for fragment size distribution by capillary electrophoresis. Library construction and sequencing were performed by Novogene. Libraries were pooled and sequenced in paired-end mode on an Illumina NovaSeq X Plus platform (Illumina, Inc., San Diego, CA, USA), generating 45.6–48.6 million raw reads per sample (Table S1).

### 4.4. Read Processing, Alignment and Differential Expression

Raw reads were filtered with fastp v0.23.1 (OpenGene, Toronto, ON, USA (https://opengene.org/, accessed on 10 January 2026)). Read pairs were discarded if either read contained adapter sequence, if more than 10% of bases were ambiguous, or if more than 50% of bases had a Phred quality ≤ 5. Clean reads were aligned with HISAT2 v2.2.1 (HISAT2 project; accessed 10 January 2026) to the *S. chuatsi* genome assembly GCA_059962705.1 generated by our group, using the corresponding gene-structure annotation (https://doi.org/10.6084/m9.figshare.33202620) to enable splice-aware alignment. Reads mapping to annotated features were counted with featureCounts v2.1.1 of the Subread package (accessed 10 January 2026), yielding 34.4–38.0 million assigned reads per library across 29,908 annotated features, of which 20,838 were detected in at least one sample.

Gene-level raw integer counts were generated using featureCounts. Differential expression analysis between FM and SM was performed in DESeq2 using the raw integer count matrix, with DESeq2 median-of-ratios normalisation and Wald testing. *p* values were adjusted using the Benjamini–Hochberg procedure. FPKM values were calculated separately for descriptive assessment of gene-expression abundance and were not used as input for DESeq2. Genes with adjusted *p* ≤ 0.05 and |log_2_ fold change| ≥ 1 were considered differentially expressed.

Differential expression between FM and SM was performed with DESeq2 v1.42.0 (Bioconductor; accessed 10 January 2026) applied to the raw integer count matrix under a negative binomial generalised linear model with median-of-ratios normalisation and Wald testing; *p* values were adjusted by the Benjamini–Hochberg procedure. FPKM values were computed separately as an expression-abundance metric for descriptive purposes and were not used as input to the differential expression model. Genes with adjusted *p* ≤ 0.05 and |log_2_ fold change| ≥ 1 were classified as differentially expressed. The contrast is expressed throughout as FM versus SM, so a positive log_2_ fold change indicates higher abundance in fast-growing fish. Complete results for all annotated features are provided in Table S3.

### 4.5. Functional Enrichment and Gene Set Enrichment Analysis

GO and KEGG over-representation analyses of differentially expressed genes were performed with clusterProfiler v4.8.1, with gene-length bias correction applied for GO, and terms with Benjamini–Hochberg-adjusted *p* < 0.05 were considered significant. Gene set enrichment analysis was performed with GSEA v4.3.2 (Broad Institute) on genes ranked by differential expression. Where no term reached adjusted *p* < 0.05, the five terms with the largest |NES| are reported as exploratory.

### 4.6. Protein Extraction, Digestion and LC-MS/MS

Muscle and liver tissues were lysed in SDT buffer supplemented with DTT (D9163-25G; Sigma-Aldrich, St. Louis, MO, USA), ultrasonicated on ice using a JY92-11N ultrasonic cell disruptor (Ningbo Scientz Biotechnology Co., Ltd., Ningbo, Zhejiang, China), and centrifuged at 12,000× *g* for 15 min at 4 °C. Proteins were alkylated with iodoacetamide (I6125-25G; Sigma-Aldrich, St. Louis, MO, USA) and precipitated with acetone (11241203810051; Beijing Chemical Works, Beijing, China). For each sample, 100 µg of protein was digested with mass-spectrometry-grade trypsin (V5280; Promega Corporation, Madison, WI, USA) at an enzyme-to-substrate ratio of 1:50 for 4 h at 37 °C, desalted using C18 columns, lyophilised, and reconstituted in 0.1% LC-MS-grade formic acid (A117-50; Thermo Fisher Scientific, Waltham, MA, USA).

Two hundred nanograms of peptide per injection were analysed using a Vanquish Neo UHPLC system (Thermo Fisher Scientific, Waltham, MA, USA) coupled to an Orbitrap Astral mass spectrometer (Thermo Fisher Scientific, Waltham, MA, USA) in data-independent acquisition mode: MS1 over *m*/*z* 380–980 at a resolution of 240,000; 300 isolation windows of 2 Th at a normalised collision energy of 25%; and MS2 over *m/z* 150–2000 at a resolution of 80,000. Spray voltage was 2.0 kV, and the ion transfer tube temperature was 290 °C.

### 4.7. Proteomic Data Processing and Differential Abundance Analysis

Raw files were processed with DIA-NN v1.8.1 (DIA-NN project; accessed 20 January 2026) against a *S. chuatsi* protein database retrieved from UniProt (taxonomy identifier 119488). Trypsin was specified with cleavage after lysine and arginine and a maximum of two missed cleavages; carbamidomethylation of cysteine was set as a fixed modification and N-terminal methionine excision as variable. Identifications were filtered to a false-discovery rate below 1% at both the precursor (Global Q-value < 0.01) and protein group (PG Q-value < 0.01) levels. Quantitative values were normalised by scaling each sample to the largest per-sample total protein intensity. Missing values were not imputed, and no additional protein-level abundance or detection filter was applied.

Differential abundance between phenotypes was assessed separately in each tissue by two-tailed Student’s *t*-test on the normalised quantities, with proteins at *p* < 0.05 reported as differentially abundant. Protein-level GSEA was evaluated using Benjamini–Hochberg-adjusted *p* values. Protein–protein interaction networks were built in STRING at a minimum interaction confidence of 0.4. Because *S. chuatsi* is absent from STRING, query sequences were aligned to *Danio rerio* proteins with DIAMOND v0.9.14, and interactions were retrieved for the resulting best-matching zebrafish proteins. Subcellular localisation was annotated from available database resources. Protein-level GSEA used signed fold-change ranking as described in Section 2.5.

### 4.8. Transcriptome-Proteome Integration

Muscle differentially expressed genes and differentially abundant proteins were matched at the entity level and classified by the confidence of the assignment: exact gene-symbol matches after case normalisation; paralog or isoform matches, where the transcript and the protein belong to the same gene family but the specific paralog cannot be resolved; and description-based matches derived from multi-word annotation strings. Directional agreement was then evaluated by comparing the sign of the RNA log_2_ fold change with the sign of the protein log_2_ fold change, both expressed as FM versus SM. Because the *S. chuatsi* annotation supplies UniProt accessions without a species-specific Bioconductor annotation package, DEP accessions were resolved through the UniProt REST API supplemented by a description-based lookup. Pathway-level co-enrichment was also examined to assess concordance between transcriptomic and proteomic signals. Analyses were performed in R.

### 4.9. Statistical Analysis

All groups comprised n = 3 biological replicates, each an individual fish. Body weight and body length were compared between FM and SM by independent-samples *t*-test. Transcriptomic thresholds were adjusted *p* ≤ 0.05 with |log_2_ fold change| ≥ 1 (DESeq2, Benjamini–Hochberg); proteomic differential abundance used an uncorrected *p* < 0.05; enrichment and GSEA significance required adjusted *p* < 0.05.

## 5. Conclusions

Muscle transcriptomics and muscle and liver proteomics identified three molecular signatures that accompany within-cohort growth divergence in *S. chuatsi*. Proteasomal and peptidase transcripts were coordinately more abundant in slow-growing muscle, the proteasome being the only KEGG pathway to reach significance and the densest module of the interaction network. Oxidative phosphorylation and mitochondrial protein categories were negatively enriched in fast-growing muscle, alongside reduced carnitine O-palmitoyltransferase 1. Haemoglobin subunits were more abundant in fast-growing fish in both tissues at the protein level, while globin transcripts in muscle differed by paralog.

These signatures were observed in fish selected from the extremes of one cohort and measured at a single time point, so they describe the molecular state that accompanies a growth phenotype and not the processes that generate it. They apply to the muscle transcriptome and to the muscle and liver proteomes examined here. Proteasome subunits, mitochondrial respiratory-chain components and haemoglobin-associated proteins emerge as leading candidates for functional investigation and evaluation as growth-associated markers in mandarin fish.

## Figures and Tables

**Figure 1:**
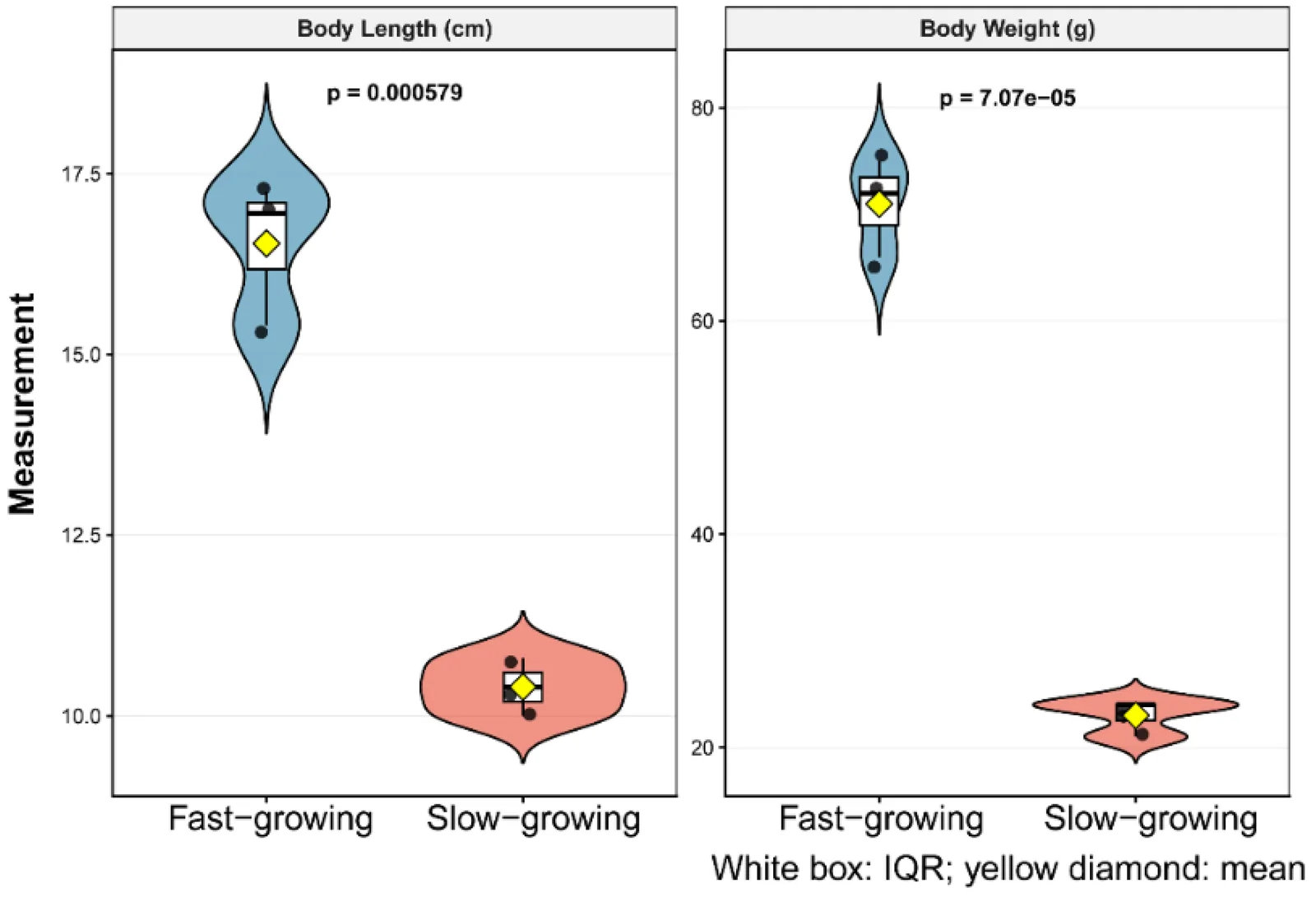
Phenotypic differences between fast-growing (FM) and slow-growing (SM) fish.

**Figure 2:**
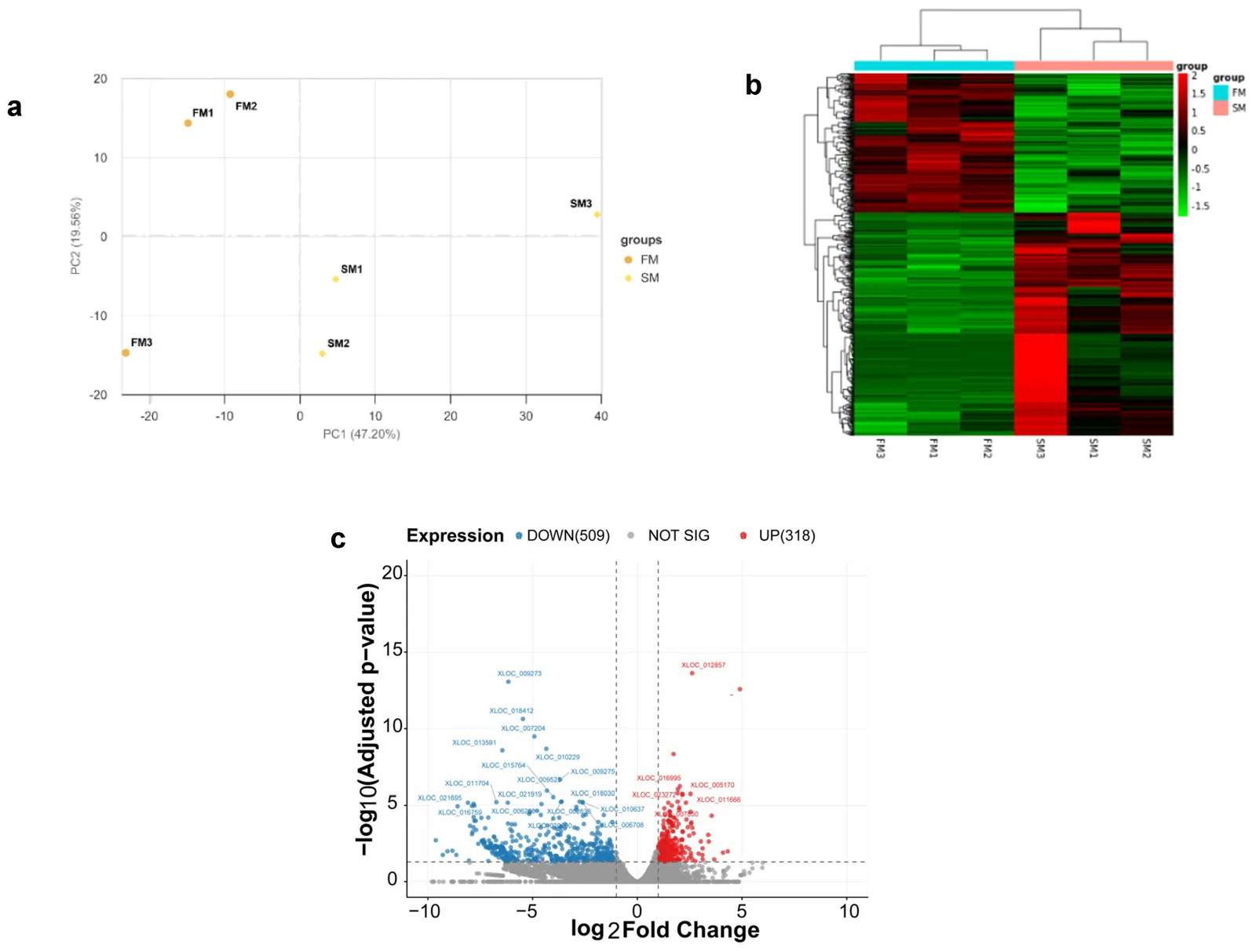
Transcriptomic differentiation between fast- and slow-growing fish. (**a**) Principal component analysis of global gene expression. (**b**) Hierarchical clustering of differentially expressed genes. (**c**) Numbers of genes with higher and lower abundance in FM relative to SM.

**Figure 3:**
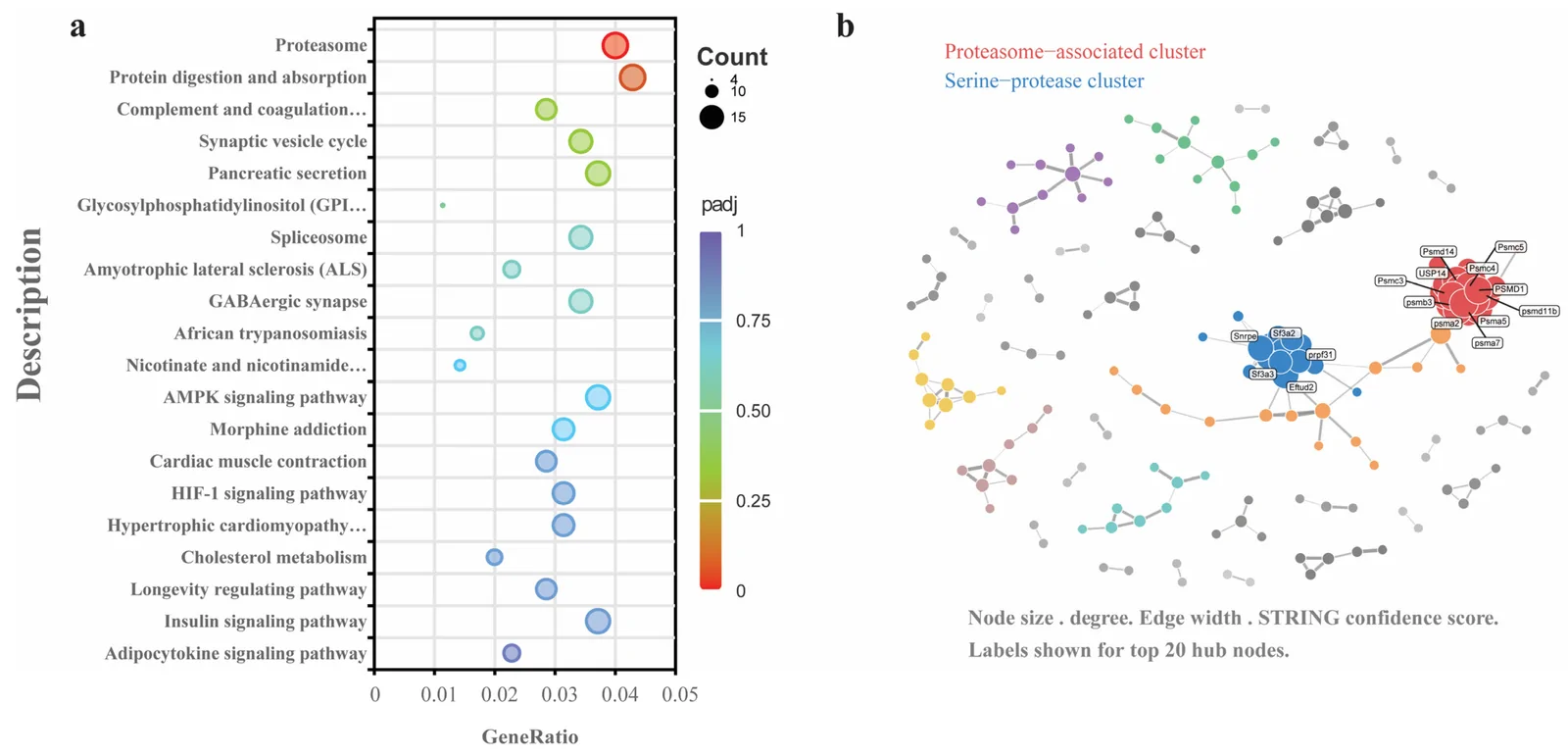
KEGG pathway and protein–protein interaction analyses of differentially expressed genes. (**a**) Significantly enriched KEGG pathways (FM versus SM). (**b**) Protein–protein interaction network (STRING confidence ≥ 0.4, Danio rerio reference).

**Figure 4:**
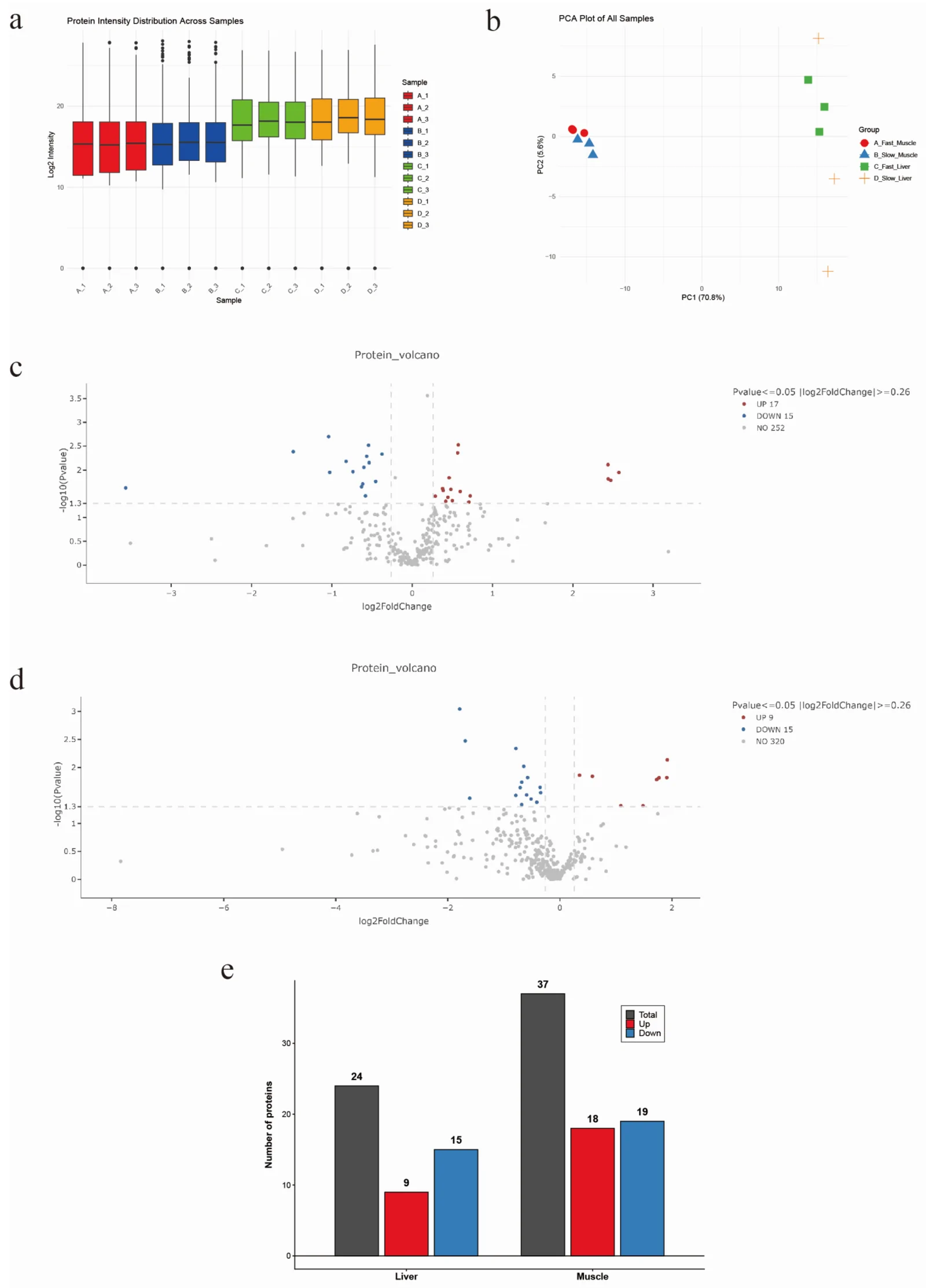
Proteomic analysis across tissues and phenotypes. (**a**) Pearson correlation heatmap and coefficient of variation distribution for the 12 samples. (**b**) Principal component analysis. (**c**–**e**) Volcano plots of differentially abundant proteins in muscle and liver (FM versus SM).

**Figure 5:**
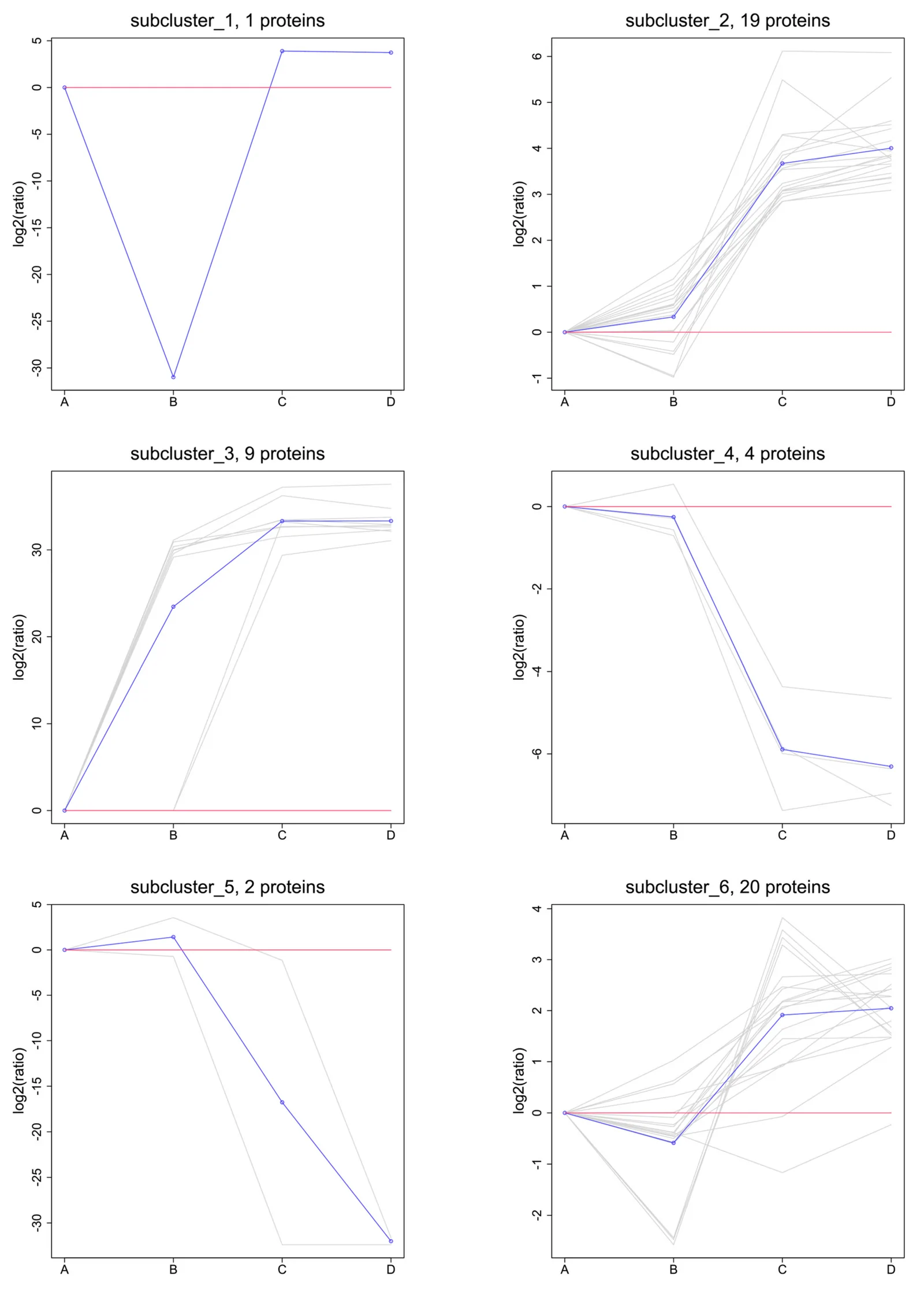
Hierarchical clustering of muscle protein abundance.

**Figure 6:**
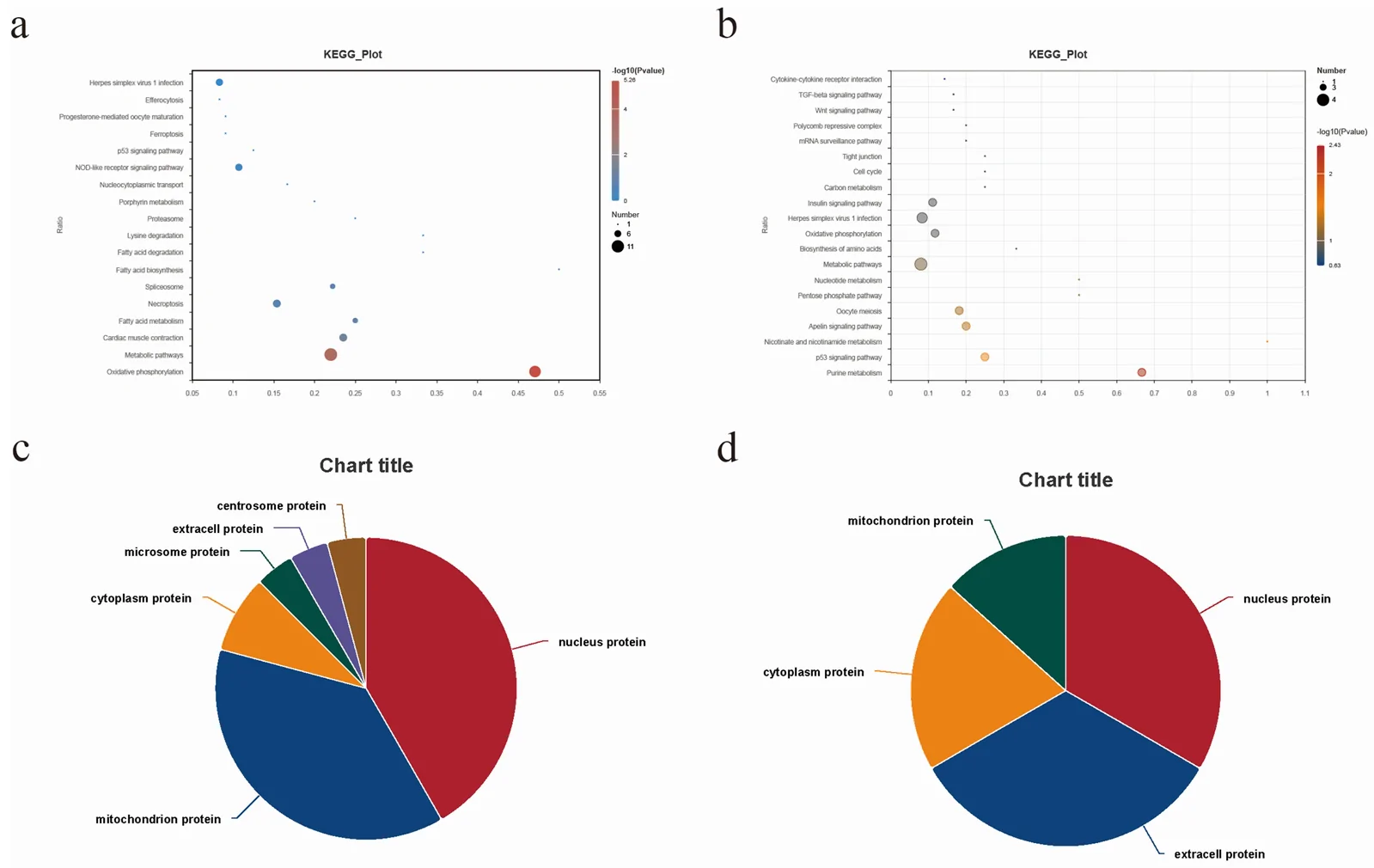
Protein-level gene set enrichment and subcellular distribution. (**a**) Muscle KEGG GSEA (FM versus SM). (**b**) Liver KEGG GSEA; no term reached adjusted *p* < 0.05. (**c**) Subcellular distribution of muscle differentially abundant proteins. (**d**) Subcellular distribution of liver differentially abundant proteins.

**Figure 7:**
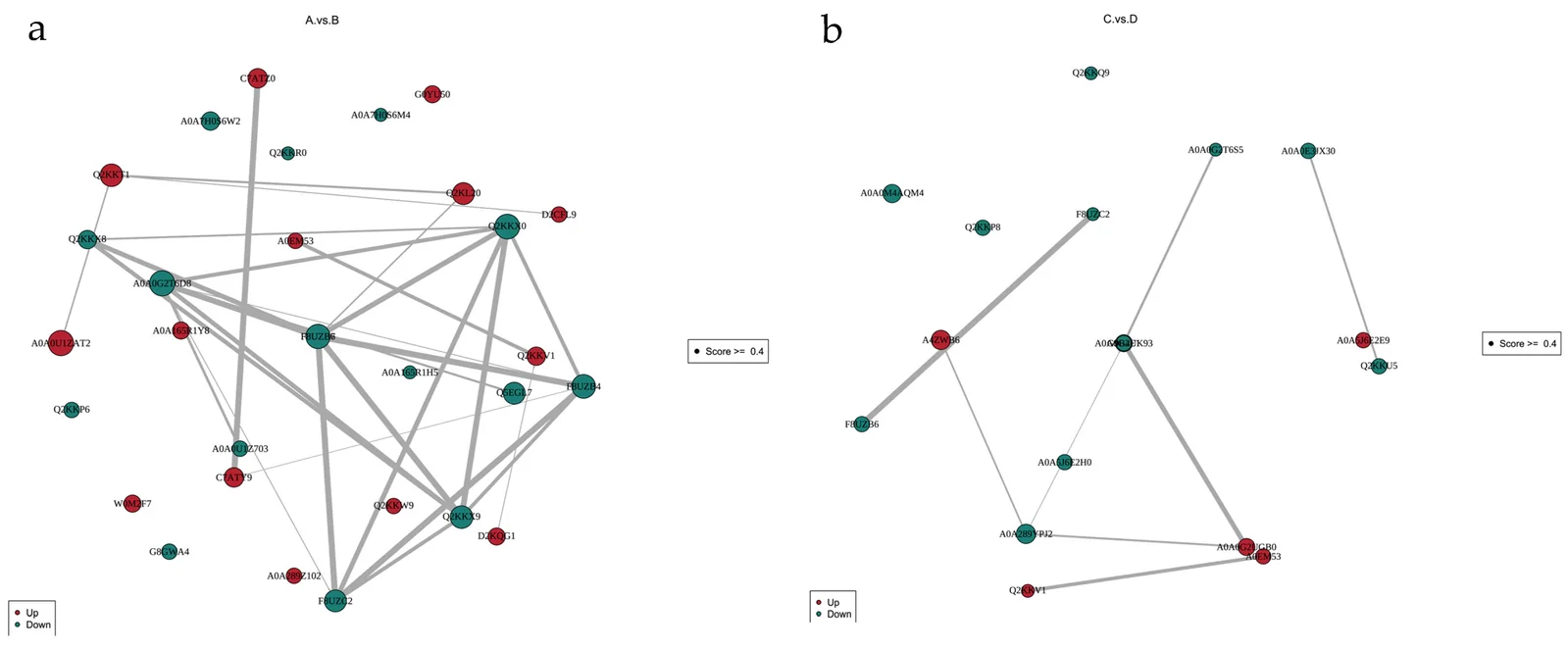
Protein–protein interaction networks of differentially abundant proteins in (**a**) muscle and (**b**) liver (STRING confidence ≥ 0.4). Node colour indicates direction (red, higher in FM; green, lower in FM); edge width is proportional to interaction confidence.

**Figure 8:**
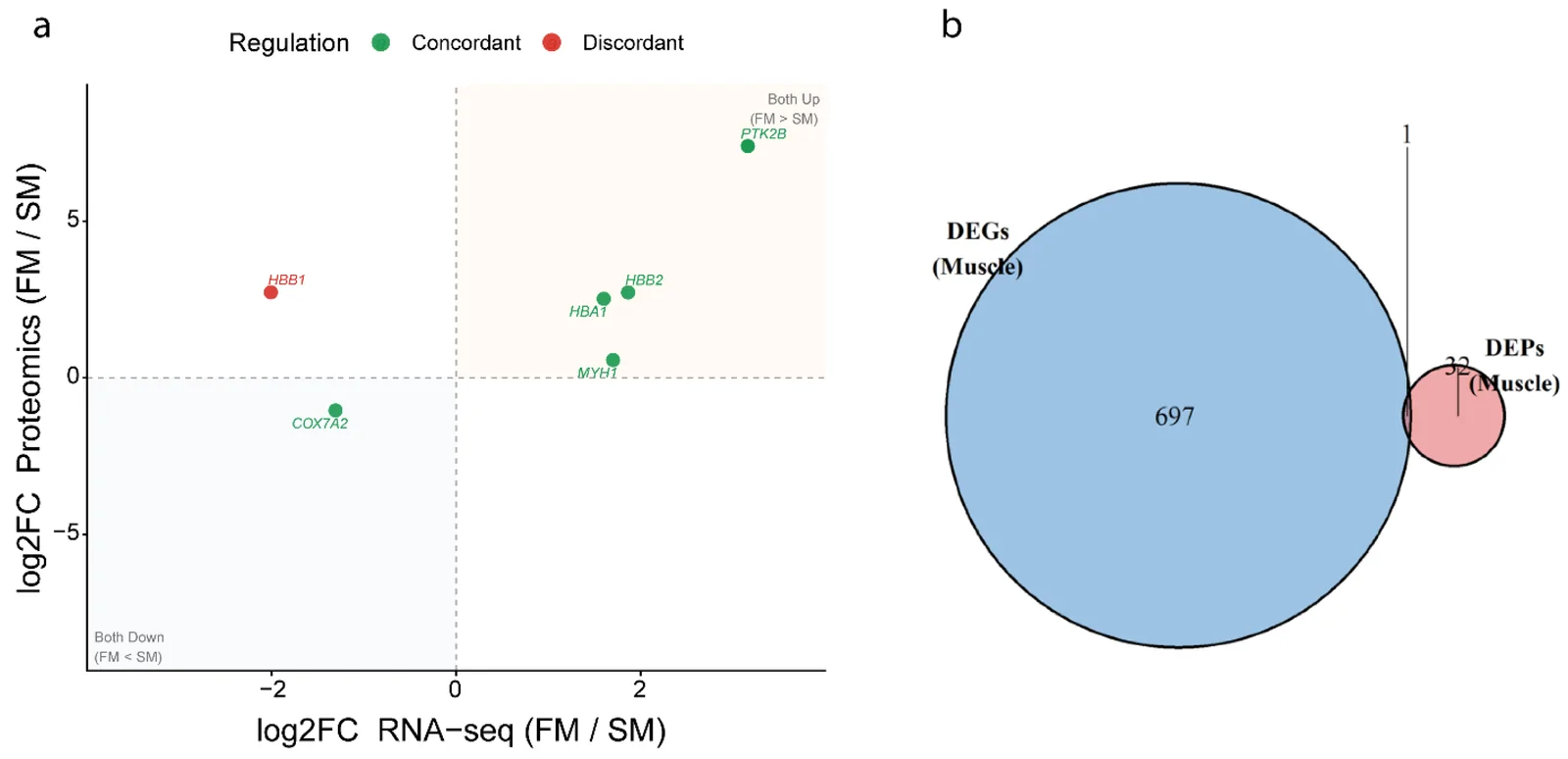
Transcript-protein correspondence in muscle. (**a**) RNA log_2_ fold change (FM/SM) against protein log_2_ fold change (FM/SM) for matched pairs, coloured by directional agreement and labelled by gene symbol. (**b**) Overlap between muscle differentially expressed genes and differentially abundant proteins at the gene-symbol level.

## Data Availability

The RNA-sequencing data generated in this study are publicly available in the NCBI Sequence Read Archive (SRA) under BioProject accession PRJNA1476567 (https://www.ncbi.nlm.nih.gov/sra/PRJNA1476567). The *S. chuatsi* genome assembly used for read mapping has been deposited in NCBI GenBank under assembly accession GCA_059962705.1. Gene structure annotation files have been deposited at Figshare (https://doi.org/10.6084/m9.figshare.33202620). Complete differential expression results are provided in Table S3. Proteomic identification and quantification results supporting the reported analyses are provided in the Supplementary Materials.

## Supplementary Materials

The following supporting information can be downloaded at https://www.mdpi.com/article/10.3390/ijms27198578/s1.
