# Supplementary material for: Integrated Multi-Omics Analysis Reveals Molecular Signatures Associated with Growth Divergence in Mandarin Fish (Siniperca chuatsi)
Source: Int J Mol Sci. 2026 Sep 25;27(19):8578. doi: 10.3390/ijms27198578 (PMC13665018; doi:10.3390/ijms27198578)
Supplement: Supplementary file 1 [file ijms-27-08578-s001.zip › ijms-4496541-Supplementary Materials.pdf]

Supplementation file

Table S1: Summary statistics of RNA sequencing data quality and read mapping across samples

Sample	Raw reads	Clean reads	Retention rate	Q20	Q30	GC	Error rate
FM1	47992806	47188652	98.3	99.3	97.3	50.4	0.01
FM2	48585340	47585650	97.9	99.3	97.2	50.1	0.01
FM3	45571780	43854290	96.2	99.3	97.2	50.4	0.01
SM1	46999906	46171644	98.2	99.3	97.2	49.3	0.01
SM2	46920416	45686924	97.4	99.3	97.2	50.6	0.01
SM3	47891266	46859496	97.8	99.3	97.1	49.2	0.01

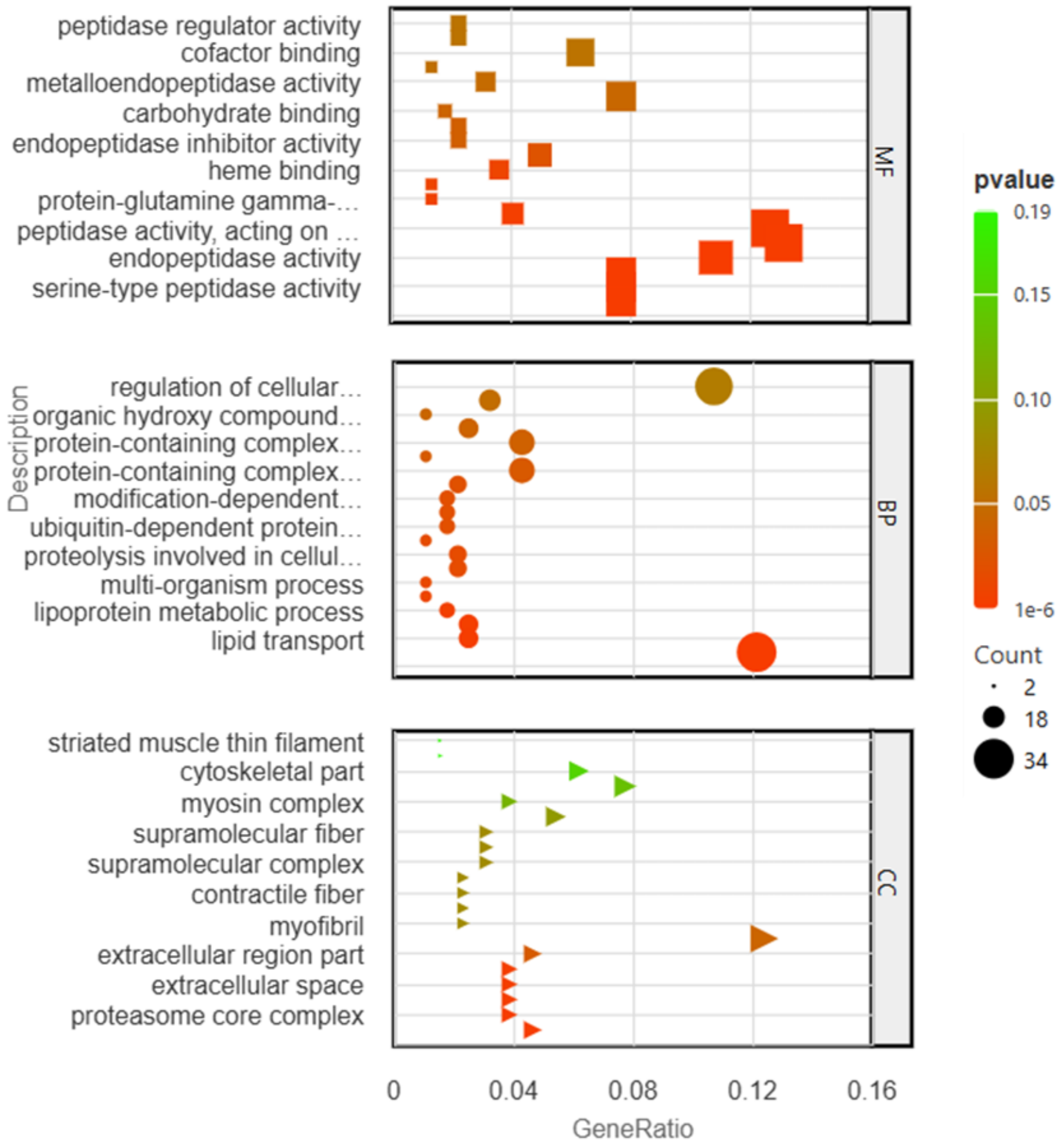

Figure S1: Gene Ontology (GO) enrichment analysis of differentially expressed genes associated with molecular function categories

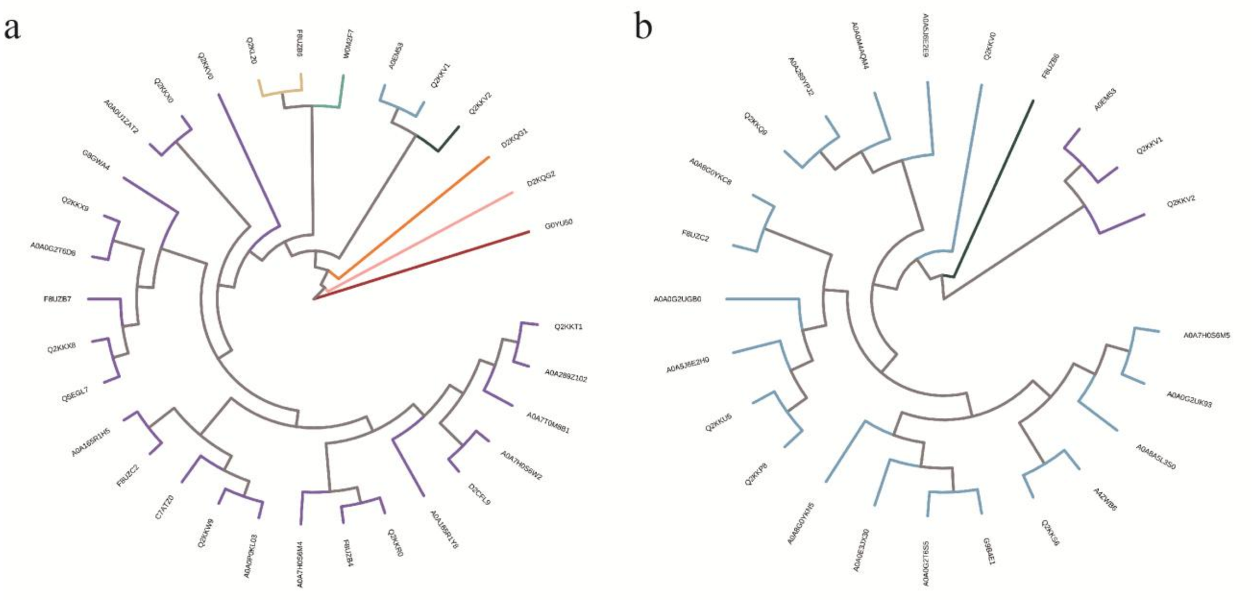

Figure S2: Differentially abundant proteins in muscle and liver.

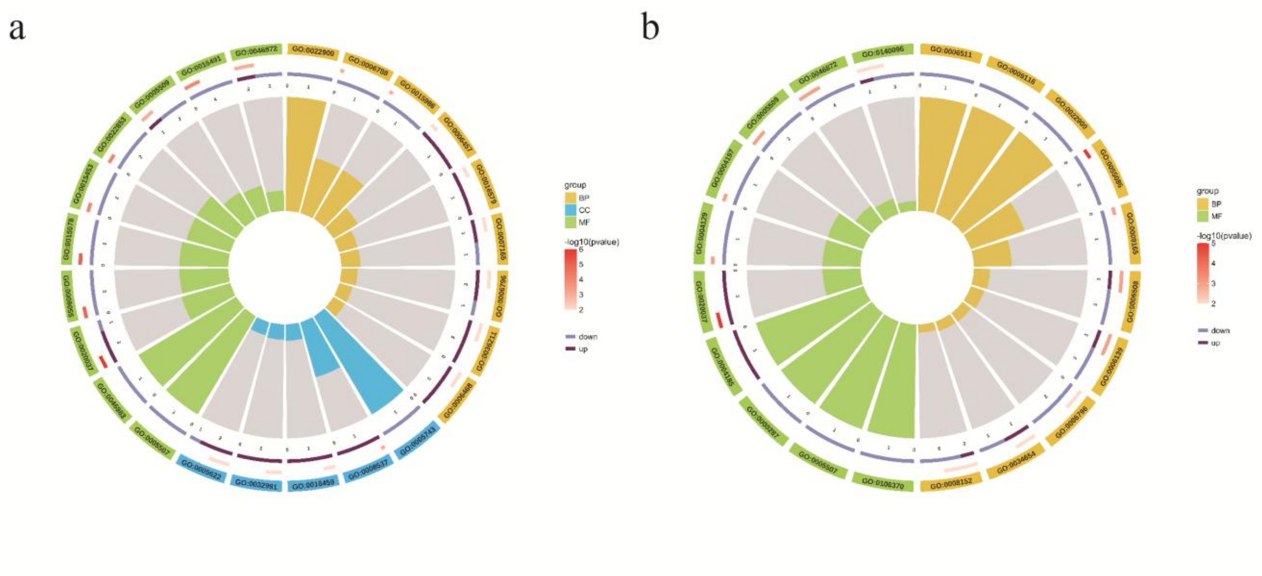

Figure S3: Gene set enrichment analysis (GSEA) of proteomic profiles in muscle tissue Two circular GO enrichment plots: (a) muscle DEPs, panel (b) liver DEPs.

Table S2: Gene-level matching between muscle differentially expressed genes (DEGs) and differentially expressed proteins (DEPs) across three sequential integration strategies.

Match_type	gene_symbol	log2FC_RNA	padj	DEP_Protein	Description	log2FC_Prot	P.Value	DEG_direction	DEP_direction
Exact_symbol	Ptk2b	3.15938773	0.04027638		Tyrosine_protein kinase	7.40070334	5.0986E-07	Up_in_FM	Up_in_FM
Paralog_isoform	hba1	1.59897391	2.4947E-05	Q2KKV0	Hemoglobin alpha_A chain (Fragment)	2.52425877	0.00288358	Up_in_FM	Up_in_FM
Paralog_isoform	hbb1	1.23932336	0.01597393	Q2KKV1	Hemoglobin beta_A chain (Fragment)	2.73111779	0.00445148	Up_in_FM	Up_in_FM
Paralog_isoform	hbb1	-2.00730669	1.3425E-05	Q2KKV1	Hemoglobin beta_A chain (Fragment)	2.73111779	0.00445148	Down_in_FM	Up_in_FM
Paralog_isoform	hbb2	1.86323776	0.00011663	Q2KKV2	Hemoglobin beta_A chain (Fragment)	2.72520447	0.00700462	Up_in_FM	Up_in_FM
Paralog_isoform	Ptk2b	3.15938773	0.04027638	C7ATY9	Tyrosine_protein kinase	7.40070334	5.0986E-07	Up_in_FM	Up_in_FM
Descriptive_name	Myosin heavy chain	-3.12155543	0.0002854	G0YU50	Fast skeletal muscle myosin heavy chain isoform 3	0.56749218	0.00341925	Down_in_FM	Up_in_FM
Descriptive_name	Myosin heavy chain	1.69973582	0.00020244	G0YU50	Fast skeletal muscle myosin heavy chain isoform 3	0.56749218	0.00341925	Up_in_FM	Up_in_FM

Descriptive_name	Myosin heavy chain	1.50562551	0.00655319	G0YU50	Fast skeletal muscle myosin heavy chain isoform 3	0.56749218	0.00341925	Up_in_FM	Up_in_FM
Descriptive_name	Cytochrome c oxidase subunit 4 isoform 2	-1.30644307	0.00017969	Q2KKX8	Cytochrome c oxidase subunit 7A2, mitochondrial (Fragment)	-1.04422714	0.00146116	Down_in_FM	Down_in_FM
Descriptive_name	Cytochrome c oxidase subunit 6A	1.3295571	0.00112384	Q2KKX8	Cytochrome c oxidase subunit 7A2, mitochondrial (Fragment)	-1.04422714	0.00146116	Up_in_FM	Down_in_FM

Table S3: Differentially abundant proteins shared between muscle and liver of fast-growing and slow-growing Siniperca chuatsi

Accession	Description	Muscle log2FC	Muscle P	Liver log2FC	Liver P	Direction
Q2KKV0	Hemoglobin alpha-A chain (fragment)	2.44	0.008	1.77	0.015	Higher in FM in both tissues
Q2KKV1	Hemoglobin beta-A chain (fragment)	2.731	0.011	1.91	0.015	Higher in FM in both tissues
Q2KKV2	Hemoglobin beta-A chain (fragment)	2.725	0.016	1.92	0.007	Higher in FM in both tissues
A0EM53	Alpha-hemoglobin A	2.44	0.015	1.73	0.016	Higher in FM in both tissues
F8UZB6	Alpha-hemoglobin A	2.22	0.013	1.83	0.005	Higher in FM in both tissues
F8UZC2	Alpha-hemoglobin A	2.16	0.01	0.25	0.0001	Higher in FM in both tissues

Note: $\log_2$FC values are expressed as FM versus SM; positive values indicate higher protein abundance in fast-growing (FM) fish. P values are from tissue-specific differential abundance analyses.
